# Reactivation or transformation? Motor memory consolidation associated with cerebral activation time-locked to sleep spindles

**DOI:** 10.1371/journal.pone.0174755

**Published:** 2017-04-19

**Authors:** Stuart Fogel, Genevieve Albouy, Bradley R. King, Ovidiu Lungu, Catherine Vien, Arnaud Bore, Basile Pinsard, Habib Benali, Julie Carrier, Julien Doyon

**Affiliations:** 1 Functional Neuroimaging Unit, Centre de Recherche de l’institut Universitaire de Gériatrie de Montréal, Montréal, Quebec, Canada; 2 Department of Psychology, University of Montreal, Montreal, Quebec, Canada; 3 School of Psychology, University of Ottawa, Ottawa, Ontario, Canada; 4 University of Ottawa Institute of Mental Health Research, University of Ottawa, Ottawa, Ontario, Canada; 5 University of Ottawa Brain & Mind Research Institute, University of Ottawa, Ottawa, Ontario, Canada; 6 Functional Neuroimaging Laboratory, INSERM, Paris, France; 7 Centre D’études Avancées en Médecine du Sommeil, Hôpital du Sacré-Cœur de Montréal, Montréal, Quebec, Canada; Tokai University, JAPAN

## Abstract

Motor memory consolidation is thought to depend on sleep-dependent reactivation of brain areas recruited during learning. However, up to this point, there has been no direct evidence to support this assertion in humans, and the physiological processes supporting such reactivation are unknown. Here, simultaneous electroencephalographic and functional magnetic resonance imaging (EEG-fMRI) recordings were conducted during post-learning sleep to directly investigate the spindle-related reactivation of a memory trace formed during motor sequence learning (MSL), and its relationship to overnight enhancement in performance (reflecting consolidation). We show that brain regions within the striato-cerebello-cortical network recruited during training on the MSL task, and in particular the striatum, were also activated during sleep, time-locked to spindles. Interestingly, the consolidated trace in the striatum was not simply strengthened, but was transformed/reorganized from rostrodorsal (associative) to caudoventral (sensorimotor) subregions. Moreover, the degree of the reactivation was correlated with overnight improvements in performance. Altogether, the present findings demonstrate that striatal reactivation linked to sleep spindles in the post-learning night, is related to motor memory consolidation.

## Introduction

Motor skills performance improves rapidly with practice. After performance levels off, motor skills can improve further during rest periods without practice; a phenomena known as “offline gains”. These gains are thought to be the result of the transformation of the memory from a fragile trace, which is susceptible to forgetting and interference; to a strengthened, interconnected, more accessible and enduring memory [1]. This process, termed memory “consolidation”, is actively enhanced by sleep [2–11], and is thought to be supported by reactivation of the memory trace. Motor skills, and in particular motor sequence learning is largely dependent on a network of brain structures including a cortico-striato-cerebellar network involving a fast learning phase, and a slow, offline phase [9,12–14]. Previous research by our group and others has shown that activity in the putamen is increased, and motor cortical [14] and cerebellar activity is decreased, during this offline period, over the course of several days [15], and more recently, this was shown to be enhanced during sleep as compared to wake [16,17]. Yet it remains a major challenge to unravel the underlying physiological mechanisms and neural substrates that support this process. Moreover, the links between physiological reactivation of the brain during sleep and behavioural memory enhancement, remain controversial.

Sleep, however, is composed of distinct stages each defined by their own set of unique physiological characteristics; including rapid eye movement sleep (REM), non-REM (NREM) sleep including NREM stage 1 (NREM1), NREM stage 2 (NREM2) and slow wave sleep (SWS). Sleep spindles are one of the prominent EEG markers, which characterize NREM2 and SWS. There is accumulating evidence that sleep spindles are related to motor skills memory consolidation [3–7]. In fact, several studies have demonstrated learning-dependent increases in sleep spindle activity following motor skill learning (MSL) [3,6,7]. Spindle activity has also been found to be correlated with both behavioral improvements [18] and sleep-dependent changes of activity in brain structures which support motor skills, such as the putamen [2,19]. These findings suggest that the striatum is functionally implicated in the consolidation of the memory trace triggered during MSL, and that spindles play a critical role in this process. Thus, sleep spindles may be markers of the reactivation of brain regions that support memory consolidation of a newly acquired sequence of motor movements. However, data supporting this view is lacking as this has been inferred based on indirect evidence. Studies to date have only reported correlations between cerebral activation using blood-oxygen-level-dependent (BOLD) functional magnetic resonance imaging (fMRI) while performing a MSL task and EEG spindle characteristics during post-learning sleep [2,19]. No studies have measured BOLD activation directly linked to spindles following procedural learning using combined EEG-fMRI. Furthermore, no direct association between reactivation during sleep and offline enhancement in motor skill performance (*i*.*e*., consolidation) has been demonstrated to date.

In addition to the aforementioned studies showing a strengthening of memory traces with time, previous work from our group suggests that BOLD activation of a motor memory trace evolves during practice [13,20] and over time [21], and thus, may not involve a simple strengthening of the same areas initially activated during learning, such as the putamen. Indeed, this work suggests that a neuroanatomical-functional shift may take place over the course of consolidation whereby activity in the more anterior dorsal (associative) part of the putamen decreased with practice, while activation in the more posterior ventrolateral (sensorimotor) part of the putamen increased during the first training session and remained elevated over a period of several days [21]. This suggests that consolidation may involve not only strengthening of activity of the putamen, but also transformation within the putamen by a shift from associative to sensorimotor regions. Evidence from humans about the role of sleep in this process is limited. However, interestingly, animal studies have shown that the ventral striatal neurons fire correlated with reward learning [22], and that reactivation of this circuit occurs offline during subsequent NREM sleep [23,24]. In addition, correlated firing between the ventral striatum and the hippocampus is observed following reward-related behavior, and an increase in the mean firing rate of the ventral striatum is associated with hippocampal ripples [25]. In addition, hippocampal ripples are time-locked to sleep spindles [26], play a causal role in memory consolidation [27] and have been suggested to be a putative mechanism for sleep-dependent reactivation and consolidation of memory traces [28]. Indeed, a network of brain structures including the hippocampus, prefrontal cortex and the ventral striatum, is involved in the goal-directed, on-line choice, action control and learning [29], faculties which are required for motor sequence learning in humans. Thus, the transformation from the early online phase of motor sequence learning which is dependent on the associative rostrodorsal subregion to the more caudoventral subregion of the striatum, emerges after a period of offline consolidation and animal studies suggest that this process may be associated with the features of sleep, such as spindles. However, the role of sleep spindles in any such transformation in humans remains to be explored. Here, we investigated whether there was evidence of *reactivation* of task-relevant brain areas during sleep associated with sleep spindles in non-REM sleep, employing an event-related analysis approach using spindle onsets from simultaneous EEG-fMRI recordings acquired during sleep on post-training nights. We also investigated whether such activations were correlated with subsequent enhancements in performance the following day. We hypothesized that regions activated during MSL practice (e.g., supplementary motor area [SMA], premotor cortex [BA 6], parietal cortex [BA 7], cerebellum and striatum) would be reactivated during subsequent sleep, time-locked to spindles. This reactivation process was expected to be correlated with overnight gains in MSL performance. Furthermore, it was predicted that a strengthening of activation in task-relevant brain areas would be observed at retest the following day, and perhaps involve *transformation/reorganization* of the trace within the striatum itself, similar to that observed in previous studies [21]. This overnight strengthening of activation was also hypothesized to be correlated with overnight gains in performance and to the extent of spindle-related activation during the night. In sum, this study aimed to identify whether the reactivation of a newly formed motor memory trace is related to the occurrence of sleep spindles, and whether these reactivations are related to the emergence of offline gains in performance.

## Materials and methods

### Overall experimental design

**Fig 1:**
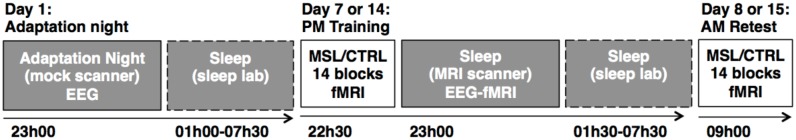
Overview of experimental design. On Day 1, subjects (n = 13, 7 female) first underwent a screening and adaptation night (bed time: 23h00) in the mock scanner under conditions similar to those experienced in both experimental and control nights to ensure that they could attain adequate sleep required for subsequent post-training sleep sessions in the MRI scanner. Subjects returned (Day 7 and Day 14) for training on either the motor sequence learning (MSL) or motor control (CTRL) task, which were administered in a random order in two separate sessions that took place on two consecutive weeks. Practice on either of these two tasks began in the evening around 22h30, and was followed by simultaneous EEG-fMRI sleep recording (starting at 23h00) lasting up to ~2.25 hours. Subjects were then allowed to sleep for the remainder of the night in the sleep lab until 07h30. This was followed by retest sessions on the same task as the previous training session (Day 8 and Day 15) comprising both behavioral and imaging data collection in the morning beginning at 09h00.

To minimize between-groups variability and utilize a parsimonious experimental approach, we employed a within-subjects design. Consolidation of the skill acquired during practice of a simple 5-item MSL task (see **Behavioral tasks and analyses**) was investigated as compared to a motor control (CTRL) task administered 1-week apart; the two task conditions being administered in a counterbalanced order (Fig 1). For both MSL and CTRL tasks, the training and retest sessions took place at 22h30 and 09h00, respectively. Immediately following the evening training session (i.e., around 23h00), post-training simultaneous EEG-fMRI was recorded while subjects were required to sleep in the scanner where spindles were subsequently identified. The sleep session was terminated when the maximum possible number of volumes for a single fMRI session (4000 volumes lasting a maximum of ~2.25 hours) in the Siemens 3.0T TIM TRIO MRI system (Siemens, Erlangen, Germany) was reached, or if subjects voluntarily terminated the session. Similar to the acclimatization night, EEG electrodes were removed after this sleep opportunity and subjects were then allowed to sleep in the nearby sleep laboratory for the remainder of the night without PSG monitoring. The retest session was administrated at 09h00, at least 1.5 hours after awakening at 07h30 to ensure the dissipation of sleep inertia. Both training and retest sessions were performed in the MRI scanner.

### Participants

A total of 30 healthy right-handed adults (20 female) aged between 20–35 years old (M = 25.6, SD = 3.6), were recruited to participate in this study, and met the screening criteria. Ethical and scientific approval was obtained from the Research Ethics Board at the “Institut Universitaire de Gériatrie de Montréal (IUGM)”, Montreal, Quebec, Canada, and informed written consent was obtained prior to entering the study.

### Screening, inclusion and exclusion criteria

Subjects were included in the study if they were right-handed [30] did not report any previous formal training as a typist or musician, nor did they consider themselves to be expert video game players. Included subjects were reportedly non-smokers, medication free, and were asked to abstain from consuming alcohol, caffeine or nicotine prior to and during the experiment. Subjects included in the study also had a normal body weight (BMI ≤ 25) and had no history of psychiatric or neurologic disorders. They all scored ≤ 8 on the Beck Depression [31] and Anxiety Inventories [32]. Participants who were categorized as extreme morning or evening types (Horne Ostberg Morningness-Eveningness Scale [33]), worked at night, or had taken a trans-meridian trip ≤ 3 months prior to the experiment, were excluded. Subjects were included if they did not exhibit signs of excessive daytime sleepiness (≤ 9 on the Epworth Sleepiness Scale [34]) and if the quality of their sleep was normal as assessed by the Pittsburgh Sleep Quality Index questionnaire [35]. Participants were required to keep a regular sleep-wake cycle (bed-time between 22h00 – 01h00, wake-time between 07h00 – 10h00) and to abstain from taking daytime naps at least 7 days prior to, and throughout their participation in the study. Compliance to the schedule was assessed using both sleep diaries and wrist actigraphy (Actiwatch 2, Philips Respironics, Andover, MA, USA) worn on the non-dominant wrist.

Each participant underwent a screening / acclimatization night beginning at 23h00, at least seven days prior to the first experimental session. Subjects were first given a two-hour opportunity to sleep in the mock scanner located at the Functional Neuroimaging Unit, Montreal, Quebec, Canada. The scanner noise and lighting conditions in the mock scanner were nearly identical to the conditions of the experimental nights in the actual MR scanner. EEG was recorded using the same MR-compatible electrode cap as during the experimental nights. To ensure that subjects were capable of falling asleep under these conditions, a minimum of five minutes of consolidated NREM sleep during the two-hour acclimatization period was required to be included in the study. All subjects were also required to sleep for at a minimum of 5 minutes of consolidated NREM sleep during both subsequent experimental nights. Following this two-hour sleep opportunity in the mock scanner, EEG electrodes were removed and subjects were allowed to sleep in the nearby sleep laboratory until 07h30. Participants slept, on average, 52.9 (+/- 24.3) minutes on the MSL night and 70.6 (+/-29.0) minutes on the CTRL night. All participants slept more than 15 minutes in total. The vast majority (90%) of sleep recordings in the MRI were longer than 20 minutes. Only two recordings were less than 20 minutes (the shortest being 16 minutes of sleep, with a total of 15 spindle events during that time, the other being 18 minutes long, with a total of 24 spindle events included in the analyses). The remaining 24 recordings included more than 30 spindle events per session in the analyses. Of the 30 subjects recruited, 6 of them did not meet the 5-minute consolidated non-REM sleep criteria (considered to be the minimum amount of data necessary for data analysis purposes) on the screening or in the MRI scanner on experimental nights. Due to the extensive protocol, seven participants voluntarily withdrew without completing the study, and 4 were excluded due to equipment malfunctions (*e*.*g*., response pad or MR scanner malfunctions). The remaining 13 subjects (M age = 27.4, SD = 3.6, 7 female) who met the inclusion criteria and completed the study were thus included in the final data analyses. This sample size, and inclusion rate, is in line with similar previous studies investigating motor learning, and sleep spindles [16,36–39], and thus have adequate statistical power for MRI and behavioural analyses.

### Behavioral tasks and analyses

#### Motor sequence learning task

Subjects were scanned while performing a motor sequence learning (MSL) task that was adapted from the sequential finger-tapping paradigm developed by Karni and colleagues [40] and coded in Cogent2000 (http://www.vislab.ucl.ac.uk/cogent.php) using MATLAB (Mathworks Inc., Sherbom, MA). A custom MR-compatible ergonomic response pad comprising four push buttons located in a horizontal row was used. First, the experimenter demonstrated the task using a slide show presentation and explained all instructions prior to participants being placed in the scanner. Once in the scanner, the session included a brief pre-training phase, where subjects were asked to repeatedly and slowly perform a 5-item sequence of finger movements (4-1-3-2-4, where 1 stands for the index finger and 4 for the little finger) using their non-dominant hand until they were able to consecutively reproduce three correct 5-item sequences. Subjects were not scanned during this pre-training, nor was it included in subsequent analyses. The pre-training procedure was simply intended to ensure that subjects had placed their hand correctly on the keypad, understood the instructions, explicitly memorized the sequence of finger movements, and could perform the 5-item sequence correctly.

For the training portion of the MSL task used in subsequent data analyses, participants were then instructed to perform the same explicitly learned sequence by tapping the finger movements as quickly as possible, while making as few errors as possible during blocks of practice. When an error occurred, participants were instructed to continue at the beginning of the 5-item sequence to ensure that practice continued in an uninterrupted fashion within each practice block. The training and retest practice sessions consisted of 14 blocks of practice (indicated by a green cross displayed in the center of the screen). Each block terminated after 60 key presses (equivalent to twelve repetitions of the sequence). Each practice block was separated by a 15 s rest period (indicated by a red cross) during which subjects were asked to keep their fingers immobile. The individual key presses were un-cued (*i*.*e*., at a self-initiated pace). Given the explicit and simple nature of the sequence, all subjects performed the sequence with a very high level of accuracy ≥ 83%, corresponding to more than 10/12 correct sequences per block. The timing of all key presses were also recorded and speed was measured by the inter-key-press interval for correct responses only. To be consistent with previous studies [2,16,41,42], gains in performance from training to retest (taken as an indicator of offline memory processing, hence consolidation) were assessed by the difference in the mean speed of the last four blocks in the training session and the mean speed of the first four blocks in the retest session.

#### Motor performance control task

The same four-button MR-safe response box was used for the CTRL task. In the MSL task, the 5-item sequence was produced by pressing each button individually, whereas in the CTRL task, all four buttons were repeatedly pressed at the same time, and at the same average pace as the MSL task. This task was designed to have the same motor performance characteristics of the MSL task (*e*.*g*., same number of finger flexion movements, same average inter-key press interval, all in the same amount of time), but importantly, without any sequence to learn and for motor movements that were self-initiated (as opposed to externally cued). Similar to the MSL task, the CTRL task was administered in two phases. Prior to being placed in the scanner, the experimenter demonstrated the task using a slide show presentation and verbally gave all instructions. Participants were then placed in the scanner. The session began with a pre-training phase where subjects were not scanned and instructed to press all four keys simultaneously following the rhythm of an auditory tone (presented monotonically at 3 Hz) as long as a green cross was displayed on the screen. This first step of the pre-training phase consisted of three blocks of practice. Each block terminated after 60 simultaneous 4-key presses. Practice blocks were separated by 15-second blocks of rest (indicated by a red cross displayed on the screen). This first pre-training step was intended to entrain subjects to the average speed of performance (~3 Hz) normally observed for the MSL task [43]. The exact same procedure was used in the second step of the pre-training phase, but this time, in the absence of the audio tones. Here, participants were instructed to maintain the same rhythm as performed in the prior three blocks of practice in the first step of the pre-training phase. The speed of performance (in Hz) was visually displayed on-screen in red text during rest blocks in order to provide feedback to the subjects on their self-initiated speed of performance. Unknown to the subjects, however, this step of the pre-training phase was terminated once performance was maintained at 3 Hz (± 0.25 Hz) for three consecutive practice blocks. This pre-training phase ensured that subjects could reliably press all four keys simultaneously at the target rhythm. Similar to the MSL task, this pre-training was not included in subsequent analyses.

For the actual practice sessions with the CTRL task, participants were instructed to follow the same rhythm as practiced during the pre-training phase, and to rest during the presentation of the red cross. Again, each of the 14 practice blocks terminated after 60 simultaneous 4-key presses, and each intervening rest period lasted 15 seconds. Similar to the MSL task, the evening CTRL training session and morning CTRL retest session were identical. To be consistent with the MSL task, performance on the CTRL task was measured as the inter-response interval between consecutive key presses (*i*.*e*., simultaneous flexion of all four fingers). The onset of the first of four finger presses was recorded and used in subsequent analyses in the event that the four fingers did not precisely touch their respective keys instantaneously.

#### Behavioral analyses

To assess the nature of the initial learning on the two tasks (MSL and CTRL) in the training session, best-fit curve estimation procedure, available in SPSS that uses least-squares regression to test the goodness of fit for linear and non-linear models [2,44] were used separately for each task to illustrate the nature of the performance changes at training and to visualize whether performance during training and retest follow the hypothesized pattern of results. In addition, a repeated-measures block (blocks 1–14) by task (MSL, CTRL) ANOVA was used to directly compare the changes in performance over the course of the training sessions. Finally, paired t-tests were used to test for offline gains in performance across the sleep interval from the end of training (mean of last 4 blocks) to the beginning of retest (mean of first four blocks) for the MSL and CTRL tasks.

### Polysomnographic recording and analysis

#### Recording parameters

EEG was recorded using an MR-compatible EEG cap (Braincap MR, Easycap, Herrsching, Germany) with 64 ring-type electrodes and two MR-compatible 32-channel amplifiers (Brainamp MR plus, Brain Products GmbH, Gilching, Germany). EEG caps included 62 scalp electrodes referenced to FCz. Two bipolar ECG recordings were taken from V2-V5 and V3-V6 using an MR-compatible 16-channel bipolar amplifier (Brainamp ExG MR, Brain Products GmbH, Gilching, Germany). Using high-chloride abrasive electrode paste (Abralyt 2000 HiCL; Easycap, Herrsching, Germany), electrode-skin impedance was reduced to < 5 KOhm. To reduce movement-related EEG artifacts, subjects' heads were immobilized in the head-coil by surrounding the subject’s head with foam cushions. EEG was digitized at 5000 samples per second with a 500-nV resolution. Data were analog filtered by a band-limiter low pass filter at 250 Hz and a high pass filter with a 10-sec time constant corresponding to a high pass frequency of 0.0159 Hz. Data were transferred via fiber optic cable to a personal computer where Brain Products Recorder Software, Version 1.x (Brain Products, Gilching, Germany) was synchronized to the scanner clock. EEG was monitored online with Brain Products RecView software using online artifact correction.

#### Preprocessing

EEG data were preprocessed by a low-pass filter (60 Hz), down-sampled to 250 samples/sec and re-referenced to averaged mastoids. Scanner artifacts were removed using the “fMRI Artifact rejection and Sleep Scoring Toolbox (FASST)” (ver. 0.302; http://www.montefiore.ulg.ac.be/~phillips/FASST.html) for MATLAB (Mathworks, Natick, Massachusetts, USA) [45], using an adaptive average subtraction method. Ballistocardiographic artifacts were then removed using an algorithm based on a combination of artifact template subtraction and event-related independent component analysis [46] for artifacts time-locked to the R-peak of the QRS complex of the cardiac rhythm.

#### Spindle detection

Following gradient artifact and ballistocardiographic artifact correction, EEG recordings were sleep stage scored according to standard criteria [47] to identify periods of NREM sleep, free of any movement artifact, during which the EEG and fMRI data were analyzed. There were no significant differences in sleep architecture or sleep spindle characteristics between the MSL and CTRL night (all p > 0.05 from paired t-tests). Sleep spindles were automatically detected at Fz, Cz and Pz (re-referenced to average mastoids) in order to include spindles from derivations where spindles are typically maximal, as described previously, using a previously published and validated method [2,48,49]. Briefly, similar to the root mean square and other related transformations commonly used to transform the raw EEG signal prior to spindle detection [50,51], this method uses a complex demodulation transformation [52] to extract the power for each data point in the frequency range of interest (*e*.*g*., 11–17 Hz). This approach has the advantage of extracting the signal of interest with a very good signal to noise ratio, and of yielding only positive data point values, hence making event detection straightforward. Also, similar to other methods that employ an individualized amplitude threshold [18] calculated from a percentile score of the whole recording (*e*.*g*., 95%), the present data from each channel and for each participant were individually normalized using a Z-score transformation derived from a 60-second sliding window to account for changes in spindle-related activity over time [53,54]. Similar to Ray et al [49], events were detected on the transformed signal using a cut-off z-score = 2.33, equivalent to the 99th percentile. We excluded any spindles that were less than 0.50 sec and included only spindles that occurred in artifact-free NREM sleep that were at least 2 seconds apart to minimize overlap in the HRF of individual spindle events.

### Brain imaging acquisition and analysis

#### Recording parameters

As described in detail previously [2] brain images were acquired using a 3.0T TIM TRIO magnetic resonance imaging system (*Siemens*, *Erlangen*, *Germany*) and a 12-channel head coil. In all subjects, a structural T1-weighted MRI image was acquired using a 3D MPRAGE sequence (TR = 2300 ms, TE = 2.98 ms, TI = 900 ms, FA = 9°, 176 slices, FoV = 256×256 mm^2^, matrix size = 256×256×176, voxel size = 1×1×1 mm^3^). Multislice T2*-weighted fMRI images were acquired during the practice sessions of the MSL and CTRL tasks with a gradient echo-planar sequence using axial slice orientation (TR = 2160 ms, TE = 30 ms, FA = 90°, 40 transverse slices, 3 mm slice thickness, 10% inter-slice gap, FoV = 220×220 mm^2^, matrix size = 64×64×40, voxel size = 3.44×3.44×3 mm^3^). Importantly, the sequence parameters were chosen so that the gradient artifact would be time stable, and the lowest harmonic of the gradient artifact (18.52 Hz) would occur outside the spindle band (11–17 Hz). This was achieved by setting the MR scan repetition time to 2160 ms, such that it matched a common multiple of the EEG sample time (0.2 ms), the product of the scanner clock precision (0.1 μs) and the number of slices (40 slices) used. Imaging parameters were the same during post-training sleep where EEG measurements were simultaneously recorded with fMRI acquisitions.

#### Image preprocessing

Functional volumes were preprocessed and analyzed using SPM8 (http://www.fil.ion.ucl.ac.uk/spm/software/spm8/; Welcome Department of Imaging Neuroscience, London, UK) implemented in MATLAB (ver. 7.14 R2012a) for OS X (Apple, Inc. Cupertino, CA). As described in detail previously [2], functional scans of each session were realigned using rigid body transformations, iteratively optimized to minimize the residual sum of squares between the first and each subsequent image separately for each session. A mean realigned image was then created from the resulting images. The structural T1-image was coregistered to this mean functional image using a rigid body transformation optimized to maximize the normalized mutual information between the two images. Coregistration parameters were then applied to the realigned BOLD time series. The coregistered structural images were segmented into grey matter, white matter and cerebrospinal fluid. An average subject-based template was created using DARTEL in SPM8. All functional and anatomical images were spatially normalized using the resulting template, which was generated from the structural scans. Finally, spatial smoothing was applied on all functional images (Gaussian kernel, 8 mm full-width at half-maximum [FWHM]).

The analysis of fMRI data, based on a mixed effects model, was conducted in 2 serial steps, accounting respectively for fixed and random effects, described in the following sections.

#### Practice sessions

For each subject, changes in brain regional responses were estimated by a model including the responses to the task (practice and rest) in each practice session (training and retest). These regressors consisted of box cars convolved with the canonical hemodynamic response function. Movement parameters were not included in the model as Johnstone and collaborators have demonstrated that it is not recommended in a block design [55]. Slow drifts were removed from the time series using a high pass filter with a cut-off period of 128 s. Serial correlations in the fMRI signal were estimated using an autoregressive (order 1) plus white noise model and a restricted maximum likelihood (ReML) algorithm. Linear contrasts were then performed for the MSL and CTRL sessions, to investigate changes in activation within each session (i.e., last 7 blocks > first 7 blocks) and to compare the difference between tasks (main effect of practice during training [MSL>CTRL]_Training_ and retest [MSL>CTRL]_Retest_ sessions). The decision to divide each 14-block practice session into 7 blocks (i.e., in half) was in order to maximize the amount of available data included in each contrast so that there was sufficient statistical power for this comparison. These linear contrasts generated statistical parametric maps [SPM(T)]. The resulting contrast images were then further spatially smoothed (Gaussian kernel 6mm FWHM) and entered in a second-level analysis, corresponding to a random effects model, hence accounting for inter-subject variance.

In the second level analyses, one-sample t-tests were performed to test the linear contrasts described above. These activation maps constituted t-statistics maps [SPM(T)] thresholded at p < 0.001 (uncorrected for multiple comparisons). All statistical inferences were performed at a threshold of p<0.001 (unc.) over the entire volume and effects significant at p<0.05, family wise error corrected over the entire volume were indicated.

#### Sleep sessions

For data acquired during the simultaneous EEG-fMRI sleep recordings, within-session series of consecutive fMRI volumes sleep stage scored as NREM sleep according to standard criteria [47] were selected from each complete fMRI time series (*e*.*g*., MSL and CTRL post-training sleep sessions). To be included in the fMRI analysis, the EEG had to be visibly movement-free and be a segment of uninterrupted NREM sleep longer in duration than 55 volumes (i.e., ~120 seconds or longer; corresponding to the minimum amount of sleep that was needed to perform the automated spindle detection), resulting in inclusion of 36% of the total recorded data (i.e., 11,466 of 31,852 MRI volumes during non-REM sleep). Each time series corresponding to NREM sleep that met these criteria were entered into the GLM as a separate session so that no gaps existed in the design matrix. For each subject, brain responses were estimated in an event-related design using a fixed-effects general linear model including responses time-locked to spindle events (11–17 Hz detected at Fz, Cz and Pz that did not overlap in time; by taking the onset for the first spindle for overlapping events). Given the limited amount of sleep, it was not possible to further subdivide spindles into slow (e.g., 11–14 Hz at Fz) and fast (e.g., 14–17 Hz at Pz) spindle types without loosing subjects due to missing data or insufficient number of spindle onsets (e.g., 30 events per spindle type, per subject, per recording). Thus, only results from full bandwidth spindles in NREM sleep were reported. Consistent with similar previous studies [36,39,56,57], the vectors including spindle events were convolved with the canonical hemodynamic response function (HRF) as well as with its temporal and dispersion derivatives. Nuisance variables in the model included the movement parameters estimated during realignment (translations in *x*, *y*, and *z* directions and rotations around *x*, *y*, and *z* axes), the squared value of the movement parameter, the first derivative of each movement parameter, and the square of the first derivative of each movement parameter, in addition to the mean white matter intensity and mean cerebral spinal fluid intensity for each subject. Slow wave activity is a defining characteristic of NREM sleep [47]. This activity was accounted for by including spectral power (μV^2^) in the delta band (0.5–4 Hz) for each TR window (2160 ms) as a variable of no interest, convolved with the hemodynamic response function. Slow drifts were removed from the time series using a high pass filter with a cut-off period of 128 s. Serial correlations in the fMRI signal were estimated using an autoregressive (order 1) plus white noise model and a restricted maximum likelihood (ReML) algorithm. Linear contrasts tested the main effect spindle events for the MSL and CTRL night and between the two post-training nights (MSL vs. CTRL) for the canonical HRF. These analyses generated statistical parametric t maps [(SPM(T)]. The resulting contrasts images were then further smoothed (FWHM 6 mm Gaussian Kernel) and entered in a second-level analysis.

The group-level analysis consisted of one sample t-tests for each contrast of interest (i.e., MSL, CTRL and MSL>CTRL contrasts). The error covariance was not assumed independent between regressors and a correction for non-sphericity was applied. To investigate the relationship between the magnitude of the spindle-dependent activation and overnight changes in performance, offline gains in performance for each subject (difference between the mean of the last 4 blocks of practice and the mean of the first 4 blocks of retest) were entered gains scores as a covariate of interest in the described GLM. These activation maps constituted maps of the t statistic [SPM(t)] testing for the main effect for each contrast of interest. All statistical inferences were performed at a threshold of p<0.001 (unc.) over the entire volume and effects significant at p<0.05, family wise error corrected over the entire volume were indicated.

#### Overlap between practice and sleep sessions

To more clearly illustrate the overlap of activations between the training and sleep sessions, the conjunction was taken as the minimum t-statistic using the global null hypothesis (see [58,59] over: 1) a t-map testing for the main effect of condition (MSL) during the training session, and 2) a t-map testing for the main effect of spindle events during the MSL night. These two statistical maps were thresholded so that when factored together, the resulting image was equivalent to a combined threshold of p < 0.001 [59]. The resulting mask image presents the areas that were consistently high and jointly activated in the training and spindle maps. It should be noted that a significant conjunction does not mean activations from both contrasts were individually significant (i.e., a conjunction of significance). Note that the minimum t-values do not have the usual Student’s t-distribution and small minimum t-values can be highly significant.

In order to investigate the degree of similarity between the training and spindle maps, we computed the spatial correlation between the two unthresholded activations maps with which the conjunction analysis was performed. The significance of the correlation was tested using a permutation-based approach from 5000 spatial permutations. This approach allows for an obtained value to be tested against a distribution of correlations generated from the permutation procedure. In addition, in order to confirm that any overlap observed in the conjunction analysis was not due to random overlap between the two maps, we generated 30 spatial permutations of randomized spindle maps. We then correlated these maps with the training map to ensure that there was no relationship between the training session and potentially random spindle-related activations (mean: r = -0.00007, p = 0.54, SD: r = 0.001, p = 0.31).

Finally, to confirm that activations time-locked to spindles and correlated with gains in performance were not simply an epiphenomena of NREM sleep, we generated the same number of random events as spindles in each segment of NREM sleep for all subjects during both the MSL night. These onsets took place between 5 and 15 seconds either before or after each originally detected spindle onset, with the condition that the random onsets did not overlap with any other spindle event. We re-ran the exact same GLM as conducted for spindle onsets, with the only difference being that the randomly generated onsets were included, as opposed to the spindle onsets.

## Results

### Sleep architecture

Subjects slept on average a total of 52.9 minutes (SD = 24.3) on the MSL night and 70.6 (SD = 29.0) on the CTRL night (Table 1). None of the subjects reached REM sleep. There were no significant differences in the duration of wake or any sleep stages (NREM1, NREM2, SWS, total NREM) between MSL and CTRL conditions. It is worth noting however that there were apparent group differences in NREM sleep from the MSL to the CTRL night. These non-statistically significant group differences were likely due to the large inter-subject variability which would be expected given the inherent limitations of sleeping in an MRI environment. Similarly, there were no differences in spindle characteristics between MSL and CTRL conditions in terms of number, density, duration, amplitude or peak frequency at Fz, Cz and Pz (Table 1).

**Table 1:** Sleep architecture and spindle results. Sleep architecture and sleep spindle parameters (mean and standard deviation) for spindles at Fz, Cz and Pz during post-training NREM sleep from EEG-fMRI recording sessions on MSL and CTRL nights.

	MSL		CTRL	
	**Sleep Architecture**			
	**Mean**	**SD**	**Mean**	**SD**
**Wake (min)**	66.9	25.3	53.2	32.4
**NREM1 (min)**	8.6	5.7	10.8	7.8
**NREM2 (min)**	34.1	18.5	42.7	21.1
**SWS (min)**	10.2	13.6	17.0	17.6
**NREM (min)**	52.9	24.3	70.6	29.0
	**Spindles Fz**			
	**Mean**	**SD**	**Mean**	**SD**
**Number**	55.15	57.32	68.00	36.60
**Density (#/min)**	0.89	0.54	0.96	0.36
**Duration (sec)**	0.91	0.35	0.84	0.33
**Amplitude (μV)**	39.76	19.87	36.84	14.63
**Frequency (Hz)**	12.96	0.76	12.90	0.74
	**Spindles Cz**			
	**Mean**	**SD**	**Mean**	**SD**
**Number**	55.61	49.91	68.46	40.57
**Density (#/min)**	0.89	0.49	0.90	0.29
**Duration (sec)**	0.95	0.40	0.90	0.37
**Amplitude (μV)**	39.77	20.43	39.44	15.81
**Frequency (Hz)**	13.58	0.64	13.53	0.63
	**Spindles Pz**			
	**Mean**	**SD**	**Mean**	**SD**
**Number**	57.77	51.60	92.85	57.87
**Density (#/min)**	0.90	0.53	1.26	0.47
**Duration (sec)**	0.98	0.40	0.95	0.37
**Amplitude (μV)**	42.69	22.07	43.84	14.86
**Frequency (Hz)**	13.54	0.62	13.49	0.63

Abbreviations: Standard deviation (SD); motor sequence learning (MSL) task; motor control (CTRL) task; slow wave sleep (SWS); non-rapid eye movement sleep (NREM); stage 1 sleep (NREM1); stage 2 sleep (NREM2). NOTE: there were no significant (p<0.05) differences (paired t-test) from MSL to CTRL for any of the above reported measures of sleep.

### Performance and offline gains in MSL

#### Training session

**Fig 2:**
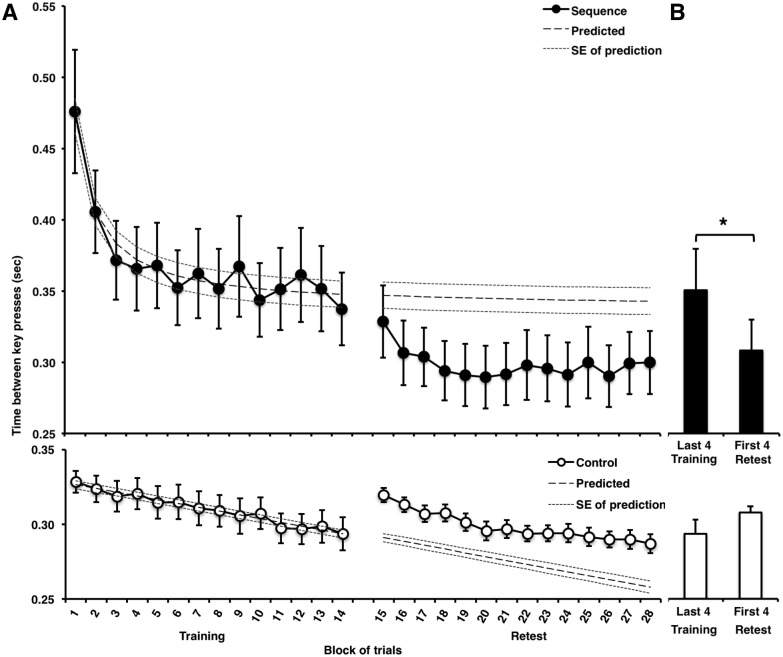
Behavioral data. **A:** Mean (+/- SEM) performance speed (inter-key press interval) during training and retest sessions for the explicit motor sequence learning (MSL) and control (CTRL) tasks. **B:** Gains in performance (reflecting consolidation) was measured using the mean (+/- SEM) inter-key-press interval in the last 4 blocks of the training *vs*. the first 4 blocks of retest. As expected, subjects showed significant gains in performance in the MSL task only (* indicates p < 0.05).

As expected, MSL performance in the training session improved rapidly across blocks of practice (Fig 2A) compared to the motor control (CTRL) task (Block [blocks 1–14] x Task [MSL, CTRL] ANOVA interaction effect: F(13,156) = 4.94, p < 0.001), and followed a prototypical learning curve (quadratic inverse function: R^2^ = 0.95, F(1,12) = 238.45, p < 0.001). By contrast, despite a gradual linear increase in speed, performance on the CTRL task did not follow the same typical learning curve (linear, straight-line function: R^2^ = 0.96, F(1,12) = 311.96, p < 0.001) during the initial training session, nor during the retest session the following day.

#### Offline gains

Accumulating evidence has shown that offline gains in explicit motor sequence performance occur in the absence of additional practice, mostly after a period of sleep [8,60,61]. Likewise, in the present study, a session (M last 4 blocks training, M first 4 blocks retest) by task (MSL, CTRL) within subjects ANOVA revealed that performance improved from training to retest in the MSL but not the CTRL task (F(1,12) = 22.65, p <0.0001). Post hoc t-tests showed that offline gains in performance on the MSL task (Fig 2B) were observed from the end of training (mean of last four blocks) to the beginning of retest (mean of first four blocks; t(12) = 3.00, p = 0.011), whereas no significant gain in performance was observed for the CTRL task (t(12) = -1.31, p = 0.214). Of note, the effect for the MSL task is significant whether as many as 7 blocks of practice are included in the means (t(12) = 3.38, p = 0.005), or as few as 3 blocks (t(12) = 2.58, p = 0.024), but not for the CTRL task. Together, these results suggest that sequence memory, but not performance on the CTRL task, was consolidated overnight.

### Online cerebral activation during MSL training and retest sessions

#### Training session

**Fig 3:**
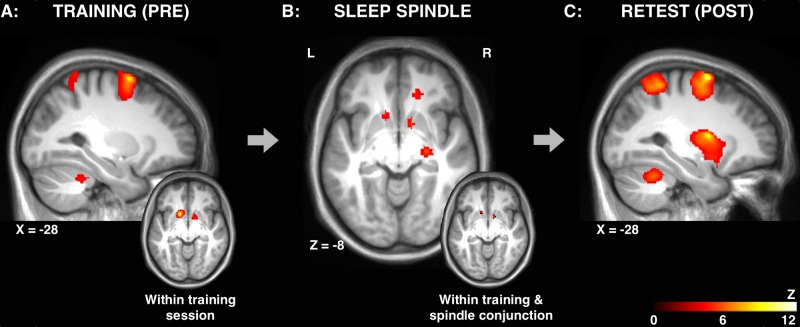
Cerebral activation during training, sleep spindles and retest. **A:** MSL-specific (MSL>CTRL) brain activation during the training session. **(inset)**: Increased activation bilaterally in the striatum from the beginning of training (first 7 blocks) as compared to the end of the training session (last 7 blocks) on the MSL task. **B:** Activations time-locked to sleep spindles following MSL. **(inset):** Conjunction of within training session-related and spindle-related activations. **C:** MSL-specific (MSL > CTRL) brain activation during the retest session. Results displayed at p<0.001, uncorrected.

Similar to previous studies [13,60], a distinct MSL-related pattern of brain activity was observed during the training session, as shown by a significant increase of activity in regions that was greater in the MSL compared to the CTRL condition (i.e., the MSL>CTRL contrast, Fig 3A**, see 2.1 in** Table 2). These included the premotor cortex (BA 6), parietal cortex (BA 7), somatosensory cortex (BA 1–3), and cerebellum (CB; lobule VI).

**Table 2:** Cerebral activations during practice sessions. Statistically significant functional imaging results from one-sample t-tests performed on training and retest sessions (see Figs 3, 4A & 5).

Hemisphere	Region	Subregion	BA	X	Y	Z	z	p
**TRAINING SESSION**								
**2.1. Main effect training session (MSL>CTRL)**								
Bilateral	Frontal	Premotor	6	-22	4	69	5.18	0.007*
Bilateral	Parietal	Precuneus	7	-2	-57	69	3.76	<0.0001
Left	Parietal	Somatosensory cortex	1–3	-44	-33	60	3.51	<0.0001
Bilateral	Cerebellum	VI		34	-46	-33	3.83	<0.0001
Right	Cerebellum	VI		21	-64	-28	3.86	<0.0001
**2.2. Within training session (MSL)**								
Bilateral	Striatum	Putamen/Caudate		-16	14	-9	5.29	0.007*
Right/Left	Parietal	Precuneus	7	-2	-66	34	3.91	<0.0001
Left	Parietal	Precuneus	7	-9	-42	76	3.19	<0.001
Right	Temporal	Angular gyrus	39	56	-63	21	3.74	<0.0001
Right	Occipital	Visual association area	18/19	30	-93	9	4.40	<0.0001
Left	Cerebellum	VI		-33	-62	-38	3.36	<0.0001
Right/Left	Brainstem	Pons		2	-36	-27	4.37	<0.0001
Bilateral	Brainstem	Midbrain		-10	-14	-16	3.72	<0.0001
**RETEST SESSION**								
**2.3. Main effect retest session (MSL>CTRL)**								
Left	Striatum	Putamen / Caudate		-26	0	12	5.13	0.009*
Bilateral	Frontal	Premotor	6	27	0	56	4.77	0.092#
Left	Frontal	Primary motor cortex	4	-62	-15	20	3.94	<0.0001
Right	Frontal	Dorsal anterior cingulate	32	8	6	48	3.45	<0.0001
Bilateral	Frontal/Parietal	Premotor / Somatosensory / Precuneus	6, 1–3, 7	-21	2	72	5.44	0.002*
Bilateral	Parietal	Precuneus	7	21	-62	70	4.52	0.077#
Left	Parietal	Supramarginal gyrus	40	-52	3	2	3.43	<0.0001
Bilateral	Cerebellum	VI		-32	-52	-30	4.14	<0.0001
Bilateral	Brainstem	Midbrain		9	-18	-6	3.62	<0.0001
**2.4. Within retest session (MSL)**								
*No significant activations*								
**RETEST-TRAINING SESSION**								
**2.5. Main effect retest-training session (MSL>CTRL)**								
Left	Striatum	Putamen / Caudate		-14	8	12	4.79	0.032*
Right	Medial Temporal	Hippocampus		15	-39	9	3.78	0.001
Right	Frontal	Supplementary motor area	6	12	-12	75	3.43	0.001
Left	Temporal	Middle temporal	21	-64	-24	-3	5.47	0.001
Left	Parietal	Somatosensory cortex	1–3	-63	-20	16	3.27	0.001

**Note:** Significant brain responses at p < 0.05 (*) and statistical trends at p < 0.10 (#) after Family Wise Error (FWE) correction over the entire volume, or over p < 0.001 uncorrected. Clusters in the same regions, less than 8mm apart are not listed.

#### Within training session

To follow-up the Increased activation of the striatum, in particular, evolved over the course of the training session (i.e., MSL>CTRL contrast in the last 7 blocks training > first 7 blocks training; Figs 3A **(inset)**, 4A and 5**, see 2.2 in** Table 2**, see** Materials and methods **for details**), suggesting that the striatum was activated more for the MSL condition in the last half of the training session, when behavioral performance became asymptotic (no significant effect of practice block at the end of training session: F(1,12) = 1.26, p = 0.29), as compared to the initial fast-learning phase, at the beginning of training period. This analysis also revealed other regions involved in MSL [21] were activated during the training session, including the parietal cortex (BA 7), cerebellum (VI) and the brainstem (e.g., pons).

**Fig 4:**
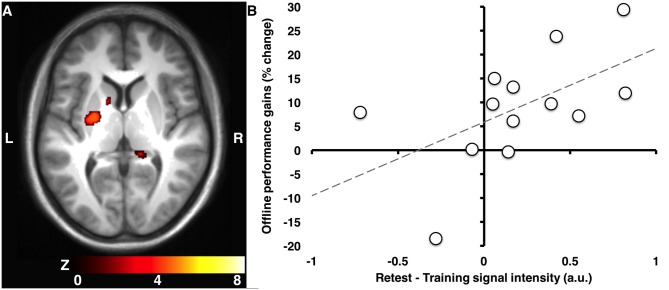
Cerebral activations during practice. Change in striatal activation from the training to the retest session in the MSL vs. CTRL condition (**A**), was associated with overnight gains in performance (**B**). Results displayed at p<0.001, uncorrected. Signal intensity expressed in arbitrary units (a.u.) obtained from raw beta weights.

**Fig 5:**
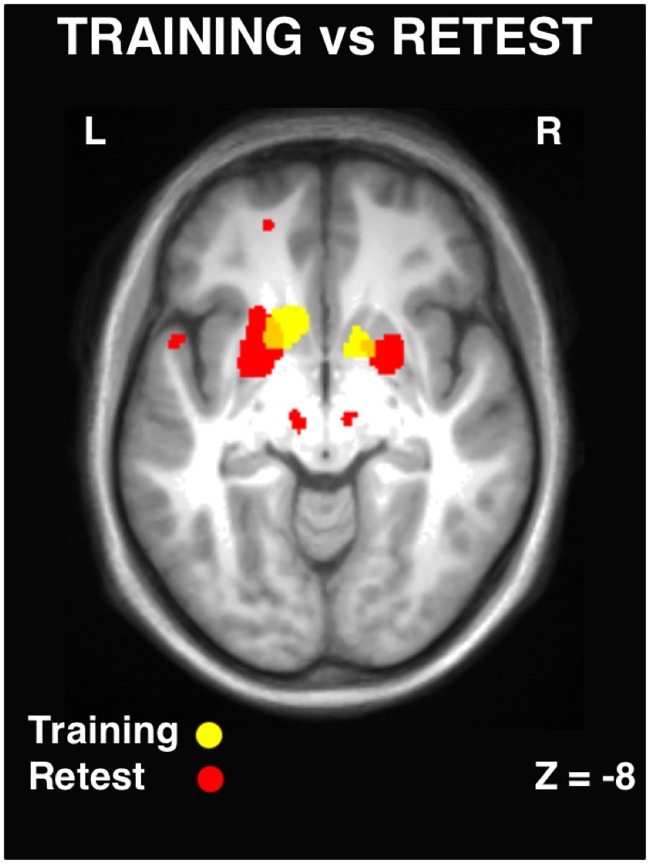
Cerebral activations during training and retest. Striatal activity within the training session (**yellow**) and the retest session (**red**). Following sleep, striatal activity was reorganized in different sub regions, as opposed to a strengthening of activity in the same regions. Results displayed at p<0.001, uncorrected.

To follow-up this analysis, we conducted a region of interest (ROI) based analysis to test the specificity of the MSL-related within session changes in the striatum. We extracted the beta values from the left and right striatum and conducted a 2 (MSL, CTRL) x 2 (first 7 blocks, last 7 blocks) repeated-measures ANOVA comparing activations in the MSL vs. CTRL condition across sessions. We observed a significant interaction in the left (F(1,12) = 5.54, p = 0.036) but not the right striatum which was greater for the MSL vs. CTRL at the end of training compared to the beginning of training. Pairwise follow-up within-session Bonferroni corrected comparisons revealed that the within-session effect was significant in the MSL but not the CTRL condition for the left (p<0.001 vs. p = 0.198, respectively) and right (p<0.001 vs. p = 0.422, respectively) striatum. Thus, suggesting that striatal activation was increased in the MSL but not the CTRL condition during the training session.

#### Retest session

After a night of sleep, during the retest session, significant motor sequence-related (MSL>CTRL contrast) activity was again observed in the same regions as the training session (e.g., BA 6, BA 7 and the cerebellum), except that sequence-specific activation (i.e., the MSL>CTRL retest contrast) of the striatum was prominently enhanced at retest during practice the following morning (see Fig 3C**, see 2.3 in** Table 2). Additional activations at retest that were not significant during the training session were also observed that support the slow phase [62] of motor learning (e.g., primary motor cortex, anterior cingulate and additional recruitment of parietal regions).

#### Within retest session

There was no change in striatal activity within the retest session itself with additional practice from the start (MSL>CTRL first 7 blocks) as compared to the end (MSL>CTRL last 7 blocks) of the retest session (**see 2.4 in** Table 2), suggesting that after sleep, the striatal memory trace was stable (i.e., consolidated).

#### Training vs. retest session

After a night of sleep, activity in the region of the striatum that was activated during the training session was significantly increased from the training to the retest session in the MSL vs. CTRL conditions (Fig 4A, **see 2.5 in** Table 2), amongst other structures such as the hippocampus, supplementary motor area and somatosensory cortex. Importantly, changes in striatal activity from training to retest were correlated with overnight gains in performance (Fig 4B**;** r(11) = 0.56, p = 0.047).

In addition, there was a reorganization of cerebral activity, particularly in the striatum, from rostrodorsal (associative) to more caudoventral (sensorimotor) regions that was remarkably similar to a previous report [21]. This reorganization took place from the training (MSL>CTRL) to the retest (MSL>CTRL) session (Fig 5; showing the overlay of both sessions). Taken together, these results suggest that sub-regions of the striatum that show increased activation during the MSL training session (as compared to the CTRL condition) are not simply increased further after a period of sleep as one might predict. Rather, there is within-striatal reorganization of activity, and the extent of changes in striatal activity is associated with MSL.

### Reactivation of brain regions recruited during training, time-locked to spindles during NREM sleep

Activations time-locked to sleep spindles during NREM sleep in the MSL condition were observed in the striatum (putamen and globus pallidum), the hippocampus, premotor area, frontal eye fields, thalamus and cerebellum (Table 3). To examine the hypothesis that regions recruited during practice of the motor sequence were reactivated again during sleep, we conducted a whole-brain spatial correlation analysis between MSL condition activation maps (i.e., *statistical map 1*: changes within MSL training session, **see 2.2** in Table 2; and *statistical map 2*: spindle-related activation during sleep following MSL training, see Table 3). Importantly, the level of activation between these two maps at the whole brain level, was significantly correlated (r = 0.15, p < 0.001), suggesting that regions with increased activation more so during training, were activated again more so during subsequent sleep, time-locked to sleep spindles.

**Table 3:** Spindle-related cerebral activations. Statistically significant functional imaging results for the main effect of activations time-locked to sleep spindles during NREM sleep in the MSL condition (see Fig 3).

Hemisphere	Region	Subregion	BA	X	Y	Z	z	p
Right	Striatum	Putamen		12	10	-8	3.95	<0.0001
Right	Striatum	Globus Pallidus		26	-18	-8	3.85	<0.0001
Left	Medial Temporal	Hippocampus		-20	-22	-28	3.46	<0.0001
Bilateral	Frontal	Premotor	6	-14	-4	76	4.22	<0.0001
Right	Frontal	Frontal eye fields	8	14	36	56	3.58	<0.0001
Right/Left	Thalamus			2	-18	17	4.67	<0.0001
Bilateral	Cerebellum	Crus 1		-42	-78	-22	5.27	0.012*
Bilateral	Cerebellum	VIII		-26	-50	-50	4.24	<0.0001
Right/Left	Cerebellum	Vermis VI, VIII		-2	-62	-46	4.26	<0.0001

**Note:** Significant brain responses at p < 0.05 after Family Wise Error (FWE) correction over the entire volume (*) or over p < 0.001 uncorrected. Clusters in the same regions, less than 8mm apart are not listed.

Moreover, a conjunction analysis between these same maps revealed that the striatum was activated bilaterally both during the training session, as well as during sleep spindles in the night immediately following the training session (Fig 3B **inset**), suggesting that spindles support reactivation of the newly formed memory trace. However, the possibility remained that the overlap between the training activation map and spindle-related map could be due simply to chance or inter-individual differences in global activation. To ensure that this was not the case, we conducted a conjunction analysis between the training map and randomized spatial permutations of the spindle map (see Materials and methods for details). We found no correlation between the training map and the randomized spatial permutations of spindle maps (mean: r = -0.00007, p = 0.54), hence suggesting that the increased activation within the training session was systematically related to greater reactivation of those same brain regions time-locked to sleep spindles during post-learning sleep.

Finally, to ensure that activations time-locked to spindles were specific to spindles per se, and not to some general epiphenomena of non-REM sleep, a separate analysis looked at activations time-locked to random onsets during non-REM sleep, instead of spindles themselves (see Materials and methods **for details**). This analysis revealed a single, small activation in the left parietal cortex that did not overlap with spindle-related activations and that did not correlate with performance gains the next day. This demonstrates that reactivations reported here were specifically related to spindle events, and not simply to NREM sleep in general. Taken together, these results suggest that regions supporting the memory trace formed during initial training, such as the putamen, were activated again in relation to sleep spindles during the post-training night.

### Relationship between spindle-related activation and overnight gains in performance

**Fig 6:**
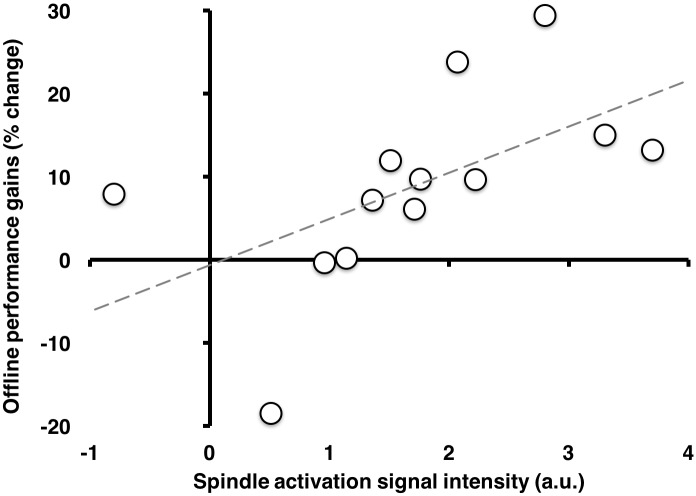
Offline gains in performance correlated with spindle activation. Spindle activation (MSL) in the ventral right striatum (putamen) correlated positively with overnight gains. Signal intensity expressed in arbitrary units (a.u.) obtained from extracting raw beta weights.

The activation of the right striatum (*n*.*b*., contralateral to the hand used to perform the MSL task) time-locked to sleep spindles recorded during post-training sleep (in the same region as presented in Figs 3 and 5 and Table 3) was correlated with overnight gains in performance (Fig 6; r(11) = 0.57, p = 0.044). This correlation remained significant even when controlling for activation in this region time-locked to spindles during the CTRL night (r(11) = 0.58, p = 0.040), thus suggesting that the relationship between post-training, spindle-related activations were specific to MSL, even when accounting for spindle-related activations in the CTRL condition.

### Relationship between spindle-related activations and practice session activations

**Fig 7:**
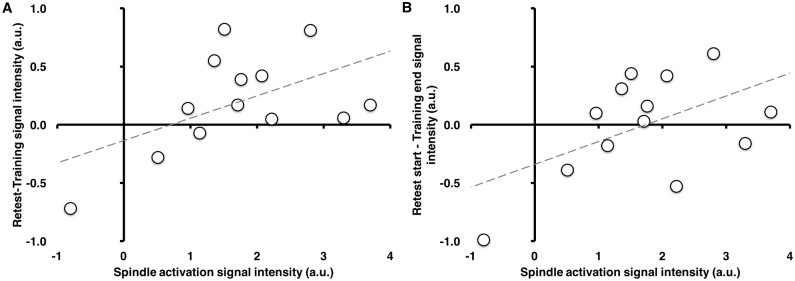
Correlation between spindle-related activation and practice session activation. **A:** Positive correlation (r(11) = 0.53, p = 0.061) between spindle-related activation in the right striatum and the change in activation from the whole training session to the whole retest session. **B:** A similar relationship (r(11) = 0.53, p = 0.065) was observed between spindle-related activations and overnight changes in the right striatum from end of training to start of retest during practice (first 7 blocks retest—last 7 blocks training). While these correlates are significant only at trend levels, note the very clear linear relationship in the scatterplots, which are consistent with our a-priori hypotheses (and would be statistically significant if a one-tailed test were employed). Signal intensity expressed in arbitrary units (a.u.) obtained from extracting raw beta weights.

While the level of striatal activity from the end of the training session to the beginning of the retest session was maintained, interestingly, spindle-related activation of the striatum tended to correlate positively with changes in striatal activation across sessions (retest vs. training), both when considering the activation over the entire testing sessions (r(11) = 0.53, p = 0.061; Fig 7A), as well as when considering within-session activation (last vs. first halves; (r(11) = 0.53, p = 0.065; Fig 7B). This pattern was maintained even when controlling for spindle-related activity during the CTRL night using partial correlations. In contrast, this pattern was not observed when using activity from the left striatum (r(11) = 0.30, p = 0.321; r(11) = 0.06, p = 0.849, respectively). This suggests that individuals who had stronger activation of the right striatum time-locked to sleep spindles during the night had a greater overnight increase in striatal activity, whereas those who had less spindle-associated activation of the striatum during sleep had a smaller overnight change in activation in this region from the end of the training session to the beginning of retest the next day.

## Discussion

**Fig 8:**
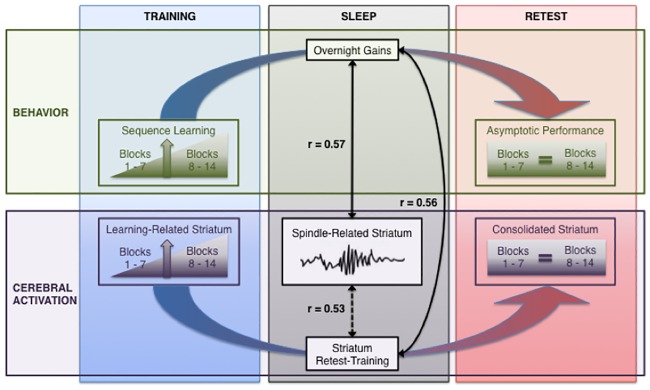
Summary of main findings. **Behavior (upper):** Sequence learning performance improved with practice over the course of the training session. Overnight gains in behavioural performance were observed from the end of the training session to the beginning of the retest session, and performance remained asymptotic at retest. **Cerebral activation (lower):** Increased learning-related cerebral activity was observed in the striatum from the beginning to the end of the training session. Reactivation of the striatum was time-locked to sleep spindles, and was correlated with offline gains in performance (r = 0.57) as well as with overnight changes in striatal activity from the Training to the Retest session (r = 0.53). The overnight change in striatal activity, which involved a transformation/reorganization within the striatum, was also correlated with offline gains in performance (r = .56). **Note:** colored arrows indicate difference measures taken between sessions to calculate overnight gains in performance and overnight changes in cerebral activity in the striatum (Retest-Training). Dashed line indicated statistical trend at p = 0.061.

Sleep has been found to be necessary for the offline enhancement of the motor memory consolidation process [2,4,8,60,61,63]. The process of strengthening newly formed memories is thought to take place via reactivation of the memory trace. Sleep spindles have been found to be involved in the consolidation process, particularly for explicit MSL, and thus may be related to the reactivation newly formed memory traces. While the brain regions that are activated by sleep spindles have been investigated [36–38], it is not known what brain regions are reactivated following new motor learning. The aim of the present study was thus to investigate whether reactivation of a motor sequence memory trace was time-locked to sleep spindles, and whether the extent of this spindle-related activation was correlated with offline gains in performance. The results of this study support four main findings: **1)** as expected, explicit motor sequence learning was associated with activity in the cortico-striatal and cortico-cerebellar systems, **2)** similar to previous reports [21], there was a transformation/reorganization of cerebral activity from the training to the retest session, particularly in the striatum, from the rostrodorsal (associative) to the more caudoventral (sensorimotor) subregion, **3)** the extent of the change in activation in the striatum was correlated with offline gains in performance, but **4)** most importantly, spindle-related reactivation of the putamen, and the extent of this reactivation was correlated with offline gains in performance as well as with the overnight change in activation of the striatum from training to retest (Fig 8).

Altogether, these results demonstrate that structures recruited during MSL, such as the striatum which exhibit increased activation from the beginning to the end of training are subsequently reactivated during the night, potentially giving rise to a reorganization of the memory trace, ultimately strengthened through activity in the striatum. This neuroanatomical-functional shift takes place during sleep, associated with spindles, whereby activity in the more anterior dorsal (associative) part of the putamen decreased with practice, while activation in the more posterior ventrolateral (sensorimotor) part of the putamen. This is consistent with previous studies showing the same shift over a period of several days [21]. Previous research by our group has shown that increased activity in the putamen is enhanced by sleep as compared to wake [16], and that activity during initial practice of a new sequence of movements is correlated with an increase in sleep spindles [18]. However, the results of the present study are important as they show, for the first time, activation of brain regions recruited during motor sequence learning were subsequently reactivated in association with sleep spindles, and that the extent of this reactivation is related to the magnitude of the enhancement in performance the following day.

Our results are complimentary in several ways to the extant animal literature, and together allow us to elucidate the underlying systems-level neurophysiology of sleep-dependent memory consolidation in humans. Previous animal studies in rodents have shown reactivation of the patterns of neural activity observed during active exploration [64–66]. This reactivation is thought to occur via the replaying of task-related hippocampal activity during post-learning NREM sleep periods. Importantly, previous work has also demonstrated that the reactivation process precedes increased firing rates in other task-relevant brain structures such as the ventral striatum [22] and prefrontal cortex [67,68], and predicts subsequent overnight memory performance [69]. Together, these rodent studies thus suggest that ventral striatal and hippocampal memory systems interact during sleep, and that they are related to offline memory enhancements. Moreover, a recent EEG-fMRI study in humans which employed a similar approach, has shown that spindle-related reactivation of the hippocampus is observed following declarative learning [39]. While we did not observe reactivation of the hippocampus, time-locked to spindles per se, the present study nevertheless show, using combined EEG-fMRI during sleep in humans, that spindle-dependent reactivation of the putamen is related to offline gains in memory performance, and importantly, that activity in the hippocampus was increased from training to retest in the MSL vs. CTRL condition, and the hippocampus was activated time-locked to sleep spindles. Interestingly, cortical replay of a motor skill was found to be time-locked to spindle events in rats [70], and invasive recording studies in humans have shown that similar to rodent studies [71,72], hippocampal “sharp wave ripples” (SWR) and spindles are temporally synchronized [26], and may facilitate the transfer of memories to the neocortex during the consolidation process [73,74]. While we do not have the means to measure SWRs directly, our results are consistent with these studies, which show that sleep spindles trigger offline reactivation of the striatum via the action spindles as well as their neocortical targets following MSL. Moreover, recent neuroimaging studies in humans have identified sleep-dependent increased activation in the hippocampus for motor sequence learning [17], particularly for the spatial (i.e., allocentric) aspect of the memory representation [75,76], for which offline gains in performance are enhanced by sleep and correlated with sleep spindles [48].

There is a large body of evidence suggesting that sleep supports the consolidation of procedural skills [3,50,60,61,77]. Moreover, the enhancement of activity in brain regions recruited during the acquisition of motor skill learning such as the striatum and hippocampus takes place after a period of sleep, but not wake [16,75,76,78], and this increased activity is correlated with performance improvements [18,19] and the features of sleep, such as spindles [19]. However, some recent studies have cast doubt as to the exact nature of the behavioural improvements, and the precise role of sleep in the consolidation process [79–83]. In the present study we observed behavioural changes that are consistent with what has been described in the literature as “offline gains” in performance, however, we cannot conclusively make the claim that these behavioural changes are greater than an equivalent period of wake. Moreover, it is important to consider that the results of the present study are purely correlational in nature and suggest that the neural changes that take place during sleep may represent sleep-related reorganization of the striatal memory trace, rather than an absolute strengthening, per se. Future research employing causal manipulations of sleep (for example see [10,11]) in combination with neuroimaging are required to understand any causal relationship between spindles and offline performance changes during sleep.

Despite furthering our knowledge of the neural and neurophysiological mechanisms mediating motor sequence memory consolidation, the present study suffers from some limitations. First, in terms of behavior, even though the magnitude of the overnight gains from the end of training to the beginning of retest is dependent on the amount of data included in the gains calculation, the computation for gains employed in this study is comparable to previous research [8,84], and remains significant when as little as 3 blocks of practice are included in the gains calculation. Second, in terms of sleep, extreme noise conditions and restricted movement in an MRI scanner makes prolonged and uninterrupted sleep less likely due to reduced subject comfort. Due to these factors, we did not anticipate subjects to easily initiate REM sleep, and thus, as expected, sleep was limited to NREM sleep only. Similarly, it was not feasible to more finely categorize spindles that occurred during NREM2 or SWS. This was due to the fact that the duration of SWS was highly variable between subjects, while being minimal or non-existent in some subjects, thus preventing reliable or valid comparisons between NREM2 and SWS spindles. A similar issue prevented us from categorizing (and eventually analyzing) spindles as slow (11–14 Hz at Fz) or fast (14–17 Hz at Pz), as some subjects had a very limited total number of spindles in NREM sleep. Finally, some degree of caution should be taken when interpreting these results as not all survive conservative whole-brain correction for multiple comparisons. However, there is a rich literature for which provides a solid rationale for specific a-priori hypotheses we have provided, and our analytics strategy is largely based on a hypothesis-driven region of interest approach. Despite that our results are consistent with, replicate and extend previous studies, further replication of these specific results would provide further support for the role of spindles in the reactivation and transformation of motor skill memory.

In summary, our results demonstrate a direct link between sleep spindles, the offline reactivation of key brain regions (the striatum, in particular) that support motor sequence memory and offline performance improvements. This represents a significant advancement in the understanding of the processes involved in motor memory consolidation, providing direct support for the hypothesis that sleep-dependent memory consolidation takes place as a result of the reactivation of brain regions recruited during initial learning. However, the resulting consolidated memory trace in the striatum is not due to a one-to-one strengthening of activity in the same region, but rather, a transformation/reorganization to overlapping areas that appears to take place as a result of spindle-related reactivation during post-training sleep.
